# Phenotypic and Functional Properties of Human Steady State CD14^+^ and CD1a^+^ Antigen Presenting Cells and Epidermal Langerhans Cells

**DOI:** 10.1371/journal.pone.0143519

**Published:** 2015-11-25

**Authors:** Cynthia. M. Fehres, Sven C. M. Bruijns, Brigit N. Sotthewes, Hakan Kalay, Lana Schaffer, Steven R. Head, Tanja D. de Gruijl, Juan J. Garcia-Vallejo, Yvette van Kooyk

**Affiliations:** 1 Department of Molecular Cell Biology and Immunology, VU University Medical Center, Amsterdam, The Netherlands; 2 DNA Array Core Facility, The Scripps Research Institute, La Jolla, CA, United States of America; 3 Department of Medical Oncology, VU University Medical Center, Amsterdam, The Netherlands; University of Bergen, NORWAY

## Abstract

Cutaneous antigen presenting cells (APCs) are critical for the induction and regulation of skin immune responses. The human skin contains phenotypically and functionally distinct APCs subsets that are present at two separated locations. While CD1a^high^ LCs form a dense network in the epidermis, the CD14^+^ and CD1a^+^ APCs reside in the dermal compartment. A better understanding of the biology of human skin APC subsets is necessary for the improvement of vaccine strategies that use the skin as administration route. In particular, progress in the characterization of uptake and activatory receptors will certainly improve APC-targeting strategies in vaccination. Here we performed a detailed analysis of the expression and function of glycan-binding and pattern-recognition receptors in skin APC subsets. The results demonstrate that under steady state conditions human CD1a^+^ dermal dendritic cells (DCs) were phenotypically most mature as measured by the expression of CD83 and CD86, whereas the CD14^+^ cells showed a higher expression of the CLRs DC-SIGN, mannose receptor and DCIR and had potent antigen uptake capacity. Furthermore, steady state LCs showed superior antigen cross-presentation as compared to the dermal APC subsets. Our results also demonstrate that the TLR3 ligand polyribosinic-polyribocytidylic acid (pI:C) was the most potent stimulator of cytokine production by both LCs and dDCs. These studies warrant further exploration of human CD1a^+^ dDCs and LCs as target cells for cancer vaccination to induce anti-tumor immune responses.

## Introduction

Dendritic cells (DCs) are a heterogeneous population of antigen-presenting cells (APCs) that are essential in the induction of adaptive immune responses. Monocyte-derived DCs (moDCs) have been classically used as an *in vitro* model for human DCs [1]. However, moDCs do not completely resemble steady state tissue resident DCs and are mainly characterized by an inflammatory profile that is hardly found *in vivo* [2]. Besides, the variety of DC subpopulations described in different human tissues makes it difficult for this *in vitro* model to fit all possible DC subtypes [3–5]. Because of limitations in the availability of viable APCs from human tissues, still relatively little is known about the functional and phenotypic specialization of the human APC network under steady state conditions and their transition and response towards inflammatory conditions. Amongst all organs, the skin is of particular interest, especially for its potential applications as application route for antigen-specific immunotherapy against cancer[6].

Recent studies have reported functional specializations of the APC subsets found in human skin. At least 3 distinct populations of APCs have been characterized in steady state human skin: epidermal Langerhans cells (LCs) that are characterized by high expression of CD1a, EpCAM, and langerin; and HLA-DR^+^ dermal cells, which can be further subdivided based on the expression of CD14 and CD1a [7]. Human LCs have been described to preferentially induce the differentiation of CD4^+^ T cells to a T helper 2 profile and to induce CD8^+^ T cells responses [8]. Human CD1a^+^ dDCs are phenotypically more mature than CD14^+^ cells, respond rapidly to CCL19/CCL21 by migrating to the lymph nodes and showed CD4^+^ and CD8^+^ T cell stimulating capacity [9]. In contrast, unstimulated, steady state CD14^+^ dermal cells have been described to secrete IL-10 and induce regulatory T cells (Tregs) and follicular T helper cells (Tfh) [8, 10]. Moreover, in steady state these cells showed a poor ability to stimulate allogeneic T cell proliferation [8, 11] and to migrate to lymph nodes [12]. Besides the CD14^+^ and CD1a^+^ APC subsets, a minor population of HLA-DR^+^CD141^hi^ DCs can be found in the dermis [13]. These cells are homologous to murine tissue CD103^+^ and splenic CD8^+^ DCs and are superior in cross-presentation of soluble antigens [12]. Variable expression of CD141 is also found on CD14^+^ dDCs, however, these cells lack the features of CD141^hi^ dDCs and induce Tregs via the secretion of IL-10 [10]. In addition, the human dermis also contains a network of tissue-resident CD14^+^ dermal macrophages, which are not able to spontaneously migrate from skin explants ex vivo [12].

Thus, skin-resident APC subsets play an important role in the polarization of T cell responses and the maintenance of peripheral tolerance via the induction of Tregs. The ability of cutaneous APCs to induce specific T cell responses can be influenced by maturation signals that these cells receive at the time of antigen recognition [8]. Under inflammatory conditions, such as in psoriasis or atopic dermatitis, skin APC numbers, and in particular CD1a^+^ dDCs, are increased, as well as their maturation status [14]. On the other hand, intradermal administration of the anti-inflammatory cytokine IL-10 increased the migration of CD14^+^CD141^+^ dermal APCs from ex vivo human skin explants. These cells expressed low levels of activatory co-stimulatory molecules, high expression of PD-L1, induced the differentiation of Tregs and, consequently, mediated a poor expansion of CD4^+^ and CD8^+^ T cells [15]. We have previously reported that skin APC subsets hardly mature upon intradermal vaccination with TLR ligands at concentrations that induce the maturation of moDCs *in vitro*, suggesting that the skin microenvironment favors the maintenance of an anti-inflammatory milieu that may be part of global peripheral tolerance mechanisms [16]. Although several studies have contributed to a better understanding of human skin-resident DC subsets, still questions remain with regard to their detailed phenotypical and functional properties in steady state and their response to inflammatory cues. In this study, we aimed to characterize the glycan-binding receptor and TLR-associated phenotype and related functional properties of human LCs and dDCs under steady state and analyzed how they are affected by inflammatory conditions.

## Material and Methods

### Reagents

rhGM-CSF and rhIL-4 were obtained from Biosource (Camarillo, CA) and used at concentrations of 262.5 U/ml and 112.5 U/ml, respectively. Polyinosinic:polycytidylic acid (pI:C) was used at a concentration of 20 ug/ml (Invivogen) and LPS (Sigma-Aldrich) at 20 ng/ml. These concentrations were based on optimal maturational effects on moDCs cultured *in vitro* as previously reported [17].

### Enzymatic isolation of LCs and dermal APCs

Primary human LCs and dermal APCs were isolated from human skin explants obtained within 24 h after cosmetic surgery from healthy donors and with informed consent (Bergman Clinics, Bilthoven, The Netherlands) as previously described [18]. Shortly, 5-mm thick slices of skin, containing the epidermis and dermis, were cut using a dermatome. The slices were incubated in Dispase II (1 mg/ml, Roche Diagnostics) in IMDM supplemented with 10% FCS (BioWhittaker), 50 U/ml penicillin (Lonza), 50 μg/ml streptomycin (Lonza) and 10 μg/ml gentamycin (Lonza) overnight at 4°C followed by the mechanical separation of dermis and epidermis using tweezers. The epidermis was washed in PBS, cut into small pieces and incubated in PBS containing DNase I (200 U/ml, Roche Diagnostics) and trypsin (0.05%, Invitrogen) for 30 min at 37°C. After incubation, a single cell suspension was generated using 100 μm nylon cell strainers (BD Falcon) and cells were layered on a lymphoprep gradient (1.077 g/ml; Alere Technologies AS.). An average of 1x10^4^ LCs per cm^2^ of tissue with a purity higher than 90% were obtained and characterized as CD1a^+^ langerin^+^ cells by flow cytometry as described below. After separation from the epidermis, the dermis was cut in small pieces and incubated in PBS containing collagenase (6 mg/ml, Roche) and dispase II (1 mg/ml) for 2 h at 37°C. A single cell population was generated using 100 μm nylon cell strainers. After obtaining single cells populations, LC and dDC cell suspensions were cultured in IMDM supplemented with 10% FCS, 50 U/ml penicillin, 50 μg/ml streptomycin and 10 μg/ml gentamycin. When indicated, isolated dDCs and LCs were MACS-sorted using CD1a and HLA-DR microbeads (MACS, Miltenyi Biotec, Germany) or DC subsets were FACS-sorted using a MoFlo cell sorter (Beckman Coulter) and fluorescent antibodies directed against HLA-DR (mIgG2a, clone L203), CD1a (mIgG1, clone HI149) and CD14 (mIgG2b, mMoP9; all from BD, San Jose, CA).

### Phenotypic analysis of isolated cells

Phenotypic analysis of isolated skin APCs was performed by flow cytometry. Cells were washed in PBS supplemented with 1% BSA and 0.02% NaN_3_ and incubated for 30 min at 4°C in the presence of appropriate dilutions of fluorochrome-conjugated mAbs to CD1a, CD14, langerin (mIgG1, clone DCGM4; Beckman Coulter Immunotech), CD70 (mIgG3, clone Ki-24), CD86 (mIgG1, clone 2331), HLA-DR (BD, San Jose, CA), HLA-ABC (mIgG2a, clone W6/32; ImmunoTools, Friesoythe, Germany) or CD83 (mIgG2b, clone HB15e; Beckman Coulter Immunotech), or corresponding isotype-matched control mAbs (BD, San Jose, CA). The cells were subsequently analyzed using a FACSCalibur (Becton Dickinson, San Jose, CA) and FlowJo software (Tree Star, Ashland, OR, USA).

### Cytokine ELISA

The levels of IL-1β, IL-6, IL-8, IL-10 and TNF-α in the supernatants were quantified using standard sandwich ELISA antibody pairs from Biosource following manufacturer’s instructions. Briefly, 50.000 LCs or dDCs were cultured in 100 μl medium supplemented with indicated TLR ligands in a 96-wells round bottom plate for 24 h, where after supernatant was harvested and analyzed for the presence of the abovementioned cytokines.

### Real-Time PCR

FACS-sorted human skin APC subsets were pooled from at least 4 skin donors to obtain sufficient cell numbers. Cells were lysed and mRNA was isolated using an mRNA Capture kit (Roche). cDNA was synthesized using the Reverse Transcription System kit (Promega) following manufacturer's guidelines. Oligonucleotides were designed using the Primer Express 2.0 software (Applied Biosystems) and synthesized by Invitrogen Life Technologies (Invitrogen). Real-Time PCR analysis was performed as previously described using the SYBR Green method in an ABI 7900HT sequence detection system (Applied Biosystems) [19]. GAPDH was used as an endogenous reference gene.

### Glycogene microarray analysis

Skin APC subsets were purified using MACS beads after 3 days of cell migration using CD19 and HLA-DR beads for the dermal APC sample and CD1a for the LC samples. CD19^+^ B cells were depleted from the dDC samples prior to RNA isolation. Analysis of gene expression was conducted using a custom gene microarray as previously described [20]. Briefly, RNA was extracted using the Qiagen RNeasy Mini kit and used to probe the GLYCOv4 oligonucleotide array, a custom Affymetrix GeneChip (Affymetrix, Santa Clara, CA, USA) designed for the Consortium for Functional Glycomics with approximately 1260 human probe-IDs related to glycosylation-related genes. Total RNA sample quality was checked with an Agilent Bioanalyzer (Agilent Technologies, Palo Alto, CA, USA). RNA from each preparation was labeled using the MessageAmp II-Biotin Enhanced Amplification kit (Ambion Inc., Austin, TX, USA). Hybridization and scanning of the GLYCOv4 chip were performed according to the Affymetrix recommended protocols [21]. Raw data files for each of the experiments performed are available at the Consortium for Functional Glycomics website (www.functionalglycomics.org/fg).

### Peptide synthesis

Peptides were produced by solid phase peptide synthesis using Fmoc chemistry with an automated peptide synthesizer (Protein Technologies, USA). Upon cleavage, peptides were purified by preparative HPLC to a yield higher than 95%. Peptide sequence was confirmed using electrospray mass spectrometry.

### Internalization of fluorescently labeled OVA

Enzymatically isolated skin APC subsets were cultured in a 96-well round bottom plate and indicated concentrations of Alexa Fluor 549-conjugated OVA were added to the wells. After 2 h of incubation, cells were washed extensively and internalization of OVA was directly measured using the FACS Calibur and FlowJo software. In order to discriminate internalization of OVA by the CD1a^+^ and CD14^+^ dermal cells, the subsets were stained using antibodies for CD1a and CD14.

### Antigen presentation to a human CD8^+^ T cell clone specific for GP100

A CD8^+^ T cell clone specific for GP100_280–288_ was generated and cultured as previously described [22]. 2x10^4^ MACS-isolated CD14^+^ cells, CD1a^+^ dDCs or LCs were cultured in a 96-well round bottom plate. A 25 aa long GP100 peptide containing the immunodominant GP100 epitope GP100_280–288_ or the 9 aa long immunodominant epitope was added to the isolated skin APC subsets at indicated concentrations. After 2 h of incubation, cells were extensively washed and GP100-specific CD8^+^ T cells (100.000/well in 100 μl medium) were added to the wells. After 24 h, supernatants were taken and IFN-γ levels were measured by sandwich ELISA using specific antibody pairs from Biosource.

### Statistical analysis

Results were analyzed using one-way or two-way ANOVA followed by Bonferrroni Multiple Comparison test using GraphPad Prism software (GraphPad Software, San Diego, CA). Results were considered to be significantly different when p<0.05.

## Results

### Dermal APCs and LCs have distinct patterns of CLRs and cytokine expression

**Fig 1:**
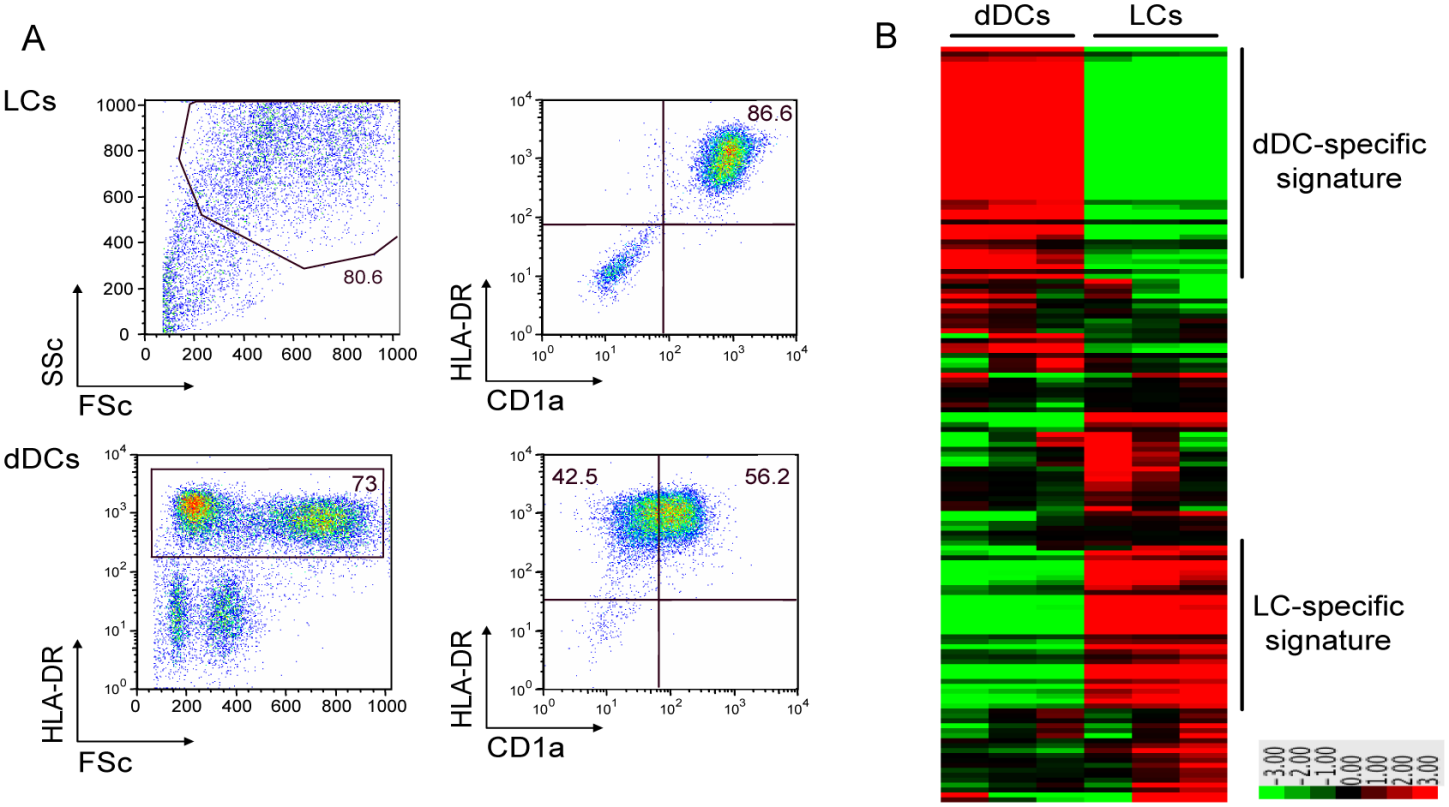
Gene expression analysis of human skin APC populations. A. LCs and dermal APCs were allowed to migrate from human skin for 3 days where after the samples were purified using CD1a and HLA-DR MACS beads, respectively. Cell purity was verified using flow cytometry. Data is shown for one representative LC and dDC sample. N = 3. B. Microarray gene array analyses on emigrated LCs and dermal APCs samples. Data are shown for 3 independent LCs and dermal APC samples.

The human skin can anatomically be divided in two main compartments: the epidermis, which contains a dense network keratinocytes and LCs; and the dermis, which is mainly composed of connective tissue with a few scattered fibroblasts, dDCs and macrophages [23]. In order to investigate the expression of genes encoding for glycan-binding receptors (C-type lectins, siglecs and galectins) in APCs present in the dermis and epidermis, we isolated APCs from both skin layers using a three days migration protocol and performed a microarray-based gene expression analysis. Prior to analysis we verified the purity of the cells by flow cytometry, as shown in Fig 1A. Isolated APCs from the dermis were purified using HLA-DR MACS beads and are therefore a heterogeneous population of cells, including the CD14^+^ dermal cells, CD1a^+^ dDCs and CD141+ dDCs. As expected, detailed analysis of the differentially expressed genes amongst both populations confirmed the exclusive expression of langerin in LCs and DC-SIGN in dermal APCs (Fig 1B). Although several genes, such as galectin-1, -3 and -9, MGL and dectin-1 were highly abundant on both APC subpopulations, a number of glycan-binding receptors defined a cell-specific expression signature as described in Table 1. Thus, next to langerin, Siglec-5 and L-SIGN were specific to LCs, whereas dermal APCs expressed higher levels of CLEC4A (also known as DCIR), Macrophage mannose receptor, AGR2, CLEC1A, COLEC12, MDL1, Galectin 2 and Siglec 7 (Table 1). Most of these glycan-binding proteins have been involved in antigen uptake, illustrating that dDC have a more specialized function in recognizing a broad repertoire of antigens for internalization than LCs. Based on these data, we can conclude that the APC subsets found in the epidermis and dermis of human skin share a significant amount of glycan-binding receptor genes, but human LC and dermal APCs also express unique CLRs illustrating that these cells may differ in their antigen recognition and uptake function.

**Table 1:** Gene expression analysis of glycan-binding receptors expressed by human LCs and dermal APCs.

dDCs					LCs				
Molecule	Sample 1	Sample 2	Average	Total score	Molecule	Sample 1	Sample 2	Average	Total score
LGALS1 (Galectin- 1)	8550,3	6605,7	7578,0	14183,7	LGALS1 (Galectin- 1)	6947,2	8344,7	7645,9	15291,9
**CLEC4A (DCIR)**	**4653,4**	**610,8**	**2632,1**	**3243,0**	**CD207 (Langerin)**	**8261,3**	**197,0**	**4229,1**	**8458,3**
LGALS3 (Galectin 3)	1952,6	1537,2	1744,9	3282,1	LGALS3 (Galectin 3)	995,1	1291,0	1143,1	2286,2
CLEC10A (MGL)	2861,0	348,5	1604,7	1953,2	LY75	380,5	1554,3	967,4	1934,8
LGALS3BP (Galectin 6 binding protein)	2675,8	66,3	1371,0	1437,3	LGALS9 (Galectin 9)	612,0	1187,4	899,7	1799,5
**DC-SIGN**	**2047,3**	**615,2**	**1331,3**	**1946,5**	LGALS3BP (Galectin 6 binding protein)	1524,9	58,3	791,6	1583,2
Siglec-1 (Sialoadhesin)	2391,9	84,8	1238,4	1323,2	CLEC10A (MGL)	921,9	40,4	481,2	962,3
LGALS9 (Galectin 9)	741,8	1172,8	957,3	2130,1	Siglec-10	744,6	72,5	408,6	817,2
CLEC7A (Dectin-1)	1568,0	307,4	937,7	1245,1	CLEC7A (Dectin-1)	554,7	71,8	313,3	626,6
**MRC1 (Macrophage mannose receptor)**	**1219,5**	**650,4**	**935,0**	**1585,4**	hCD33 (hSiglec-3)	509,3	60,7	285,0	569,9
hCD33 (hSiglec-3)	1320,1	142,8	731,5	874,2	Siglec-1 (Sialoadhesin)	381,5	75,8	228,6	457,3
**ASGR2 (Asialoglycoprotein receptor 2)**	**986,9**	**74,7**	**530,8**	**605,5**	CLEC4F [C-type lectin superfamily member 13]	184,9	185,4	185,1	370,3
CLEC4G (C-type lectin superfamily 4 member G)	148,9	858,9	503,9	1362,8	CLEC11A (Stem cell growth factor)	159,0	126,0	142,5	285,1
**CLEC1A**	**412,4**	**263,6**	**338,0**	**601,6**	Siglec14 [sialic acid binding Ig-like lectin 14]	205,4	45,8	125,6	251,2
CLEC11A (Stem cell growth factor)	481,7	169,0	325,3	494,3	**Siglec-5**	**186,3**	**33,3**	**109,8**	**219,6**
LY75 (DEC205)	169,2	394,4	281,8	676,2	Siglec-L1	149,6	37,5	93,6	187,1
CLEC4F [C-type lectin superfamily member 13]	277,8	97,3	187,5	284,8	Siglec15 [sialic acid binding Ig-like lectin 15]	70,7	60,9	65,8	131,6
Siglec-10	297,6	68,5	183,1	251,6	Siglec-8	62,9	58,8	60,8	121,7
**COLEC12 (Scavenger receptor with CTLD)**	**301,8**	**52,1**	**177,0**	**229,0**	CLECL1 (type II transmembrane protein DCAL1)	76,6	43,6	60,1	120,2
Siglec14 [sialic acid binding Ig-like lectin 14]	163,4	64,1	113,8	177,9	CLEC4G (C-type lectin superfamily 4 member G)	52,7	66,4	59,6	119,1
Siglec-9	136,8	89,5	113,2	202,7	Siglec-9	72,5	46,5	59,5	119,0
Siglec-L1	169,7	51,1	110,4	161,5	ASGR1 Asialoglycoprotein receptor 1	58,9	59,1	59,0	118,0
**CLEC5A (MDL-1)**	**155,0**	**55,9**	**105,5**	**161,4**	CLEC3B (C-type lectin domain family 3 member B)	57,9	58,8	58,4	116,7
CLECL1 (type II transmembrane protein DCAL1)	166,0	38,9	102,5	141,4	LGALS7 (Galectin 7)	45,5	67,4	56,4	112,9
**LGALS2 (Galectin 2)**	**136,9**	**53,4**	**95,1**	**148,5**	CD72	49,2	58,3	53,7	107,4
**Siglec-7**	**130,3**	**58,0**	**94,1**	**152,1**	**CLEC4M (DC-SIGNR / L-SIGN)**	**47,6**	**33,8**	**40,7**	**81,4**
ASGR1 Asialoglycoprotein receptor 1	92,6	65,0	78,8	143,8					
LGALS7 (Galectin 7)	43,4	107,6	75,5	183,1					
CLEC3B (C-type lectin domain family 3 member B)	67,2	76,0	71,6	147,6					
CD72	90,4	48,6	69,5	118,1					
Siglec-8	49,5	52,4	50,9	103,3					
Siglec15 [sialic acid binding Ig-like lectin 15]	44,2	42,1	43,1	85,2					

Average and total scores of the genes that were highly expressed are depicted for the dermal APCs samples (left columns) and LC samples (right columns). Genes exclusively expressed by either dermal APCs or LCs are depicted in bold.

### Characterization of human skin APC subsets in steady state

**Fig 2:**
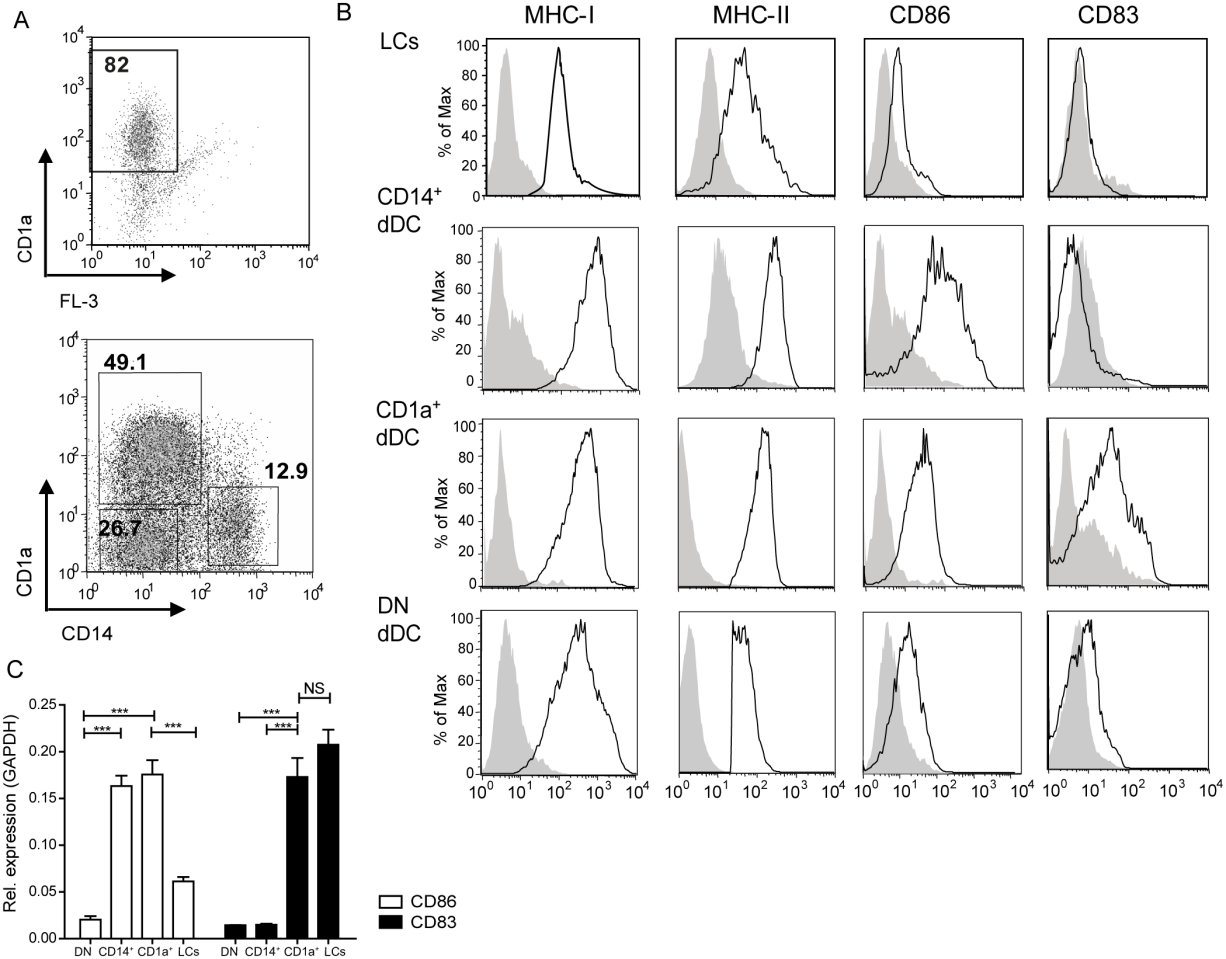
The CD1a^+^ dDC subset is the phenotypically most mature human skin APC subset under steady conditions. A. Subset distribution in percentages of CD1a^+^ and CD14^+^ dermal cells (upper panel) or CD1a^+^ LCs (lower panel) after gating on isolated HLA-DR^+^ cells. Dot plots of a representative experiment are shown. N = 8. B. Surface expression of MHC class I and II, CD86 and CD83 was measured by flow cytometry on isolated HLA-DR^+^CD14^+^ cells, HLA-DR^+^CD1a^+^ dDCs and HLA-DR^+^CD1a^high^ LCs. Grey histograms depict matching isotype controls. Histograms of a representative experiment are shown. N = 3. C. Relative mRNA levels of CD83 and CD86 compared to the housekeeping gene GAPDH are shown present in FACS-sorted, steady state DN, CD14^+^ or CD1a^+^ dermal cells and LCs. Due to low cell numbers of each subset after sorting, the subsets contain combined cells of at least 4 skin donors. Mean values ± SEM; n = 3. ***p<0.001, as measured by the one-way ANOVA followed by the Bonferroni multiple comparison test.

To further characterize the different types of APCs we performed enzymatic digestions and analyzed the resulting single cell suspensions by flow cytometry. Based on forward and side scatter properties and the expression of HLA-DR, CD1a and CD14, we characterized a CD1a^+^CD14^−^ subset, a CD14^+^CD1a^−^ subset and a double negative (DN) subset present in the dermis and the HLA-DR^+^CD1a^high^ LCs in the epidermis (Fig 2A). We first focused on the expression of activation and maturation markers and found that LCs expressed low levels of CD86 and CD83 and intermediate levels of MHC-I and MHC-II after isolation (Fig 2B). In contrast, the dDC subsets displayed higher expression levels of these molecules, specially the CD1a^+^ dDCs. In steady state, CD1a^+^ dDCs seemed activated, based on their high levels of CD86 and CD83 as compared to other dDCs. We speculated that the enzymatic treatment of the dermis and epidermis to isolate the different APC subsets might have reduced the expression of surface markers, so we sorted the abovementioned subpopulations and repeated the analysis using quantitative RT-PCR. As shown in Fig 2C, transcripts for CD86 and CD83 were highly abundant in CD1a^+^ dDCs, hardly present in DN dDCs and, in contrast to the data obtained by flow cytometry, LCs had a high expression of CD83. In summary, a side by side comparison revealed that the CD1a^+^ dDCs expresses the highest levels of maturation markers under steady state conditions as compared to DN DCs, CD14^+^ dermal cells and LCs, which did express MHC-I and –II, but varying amounts of the co-stimulatory molecules CD86 and CD83.

### Human skin APC subsets show a restricted expression pattern of CLRs and TLRs

**Fig 3:**
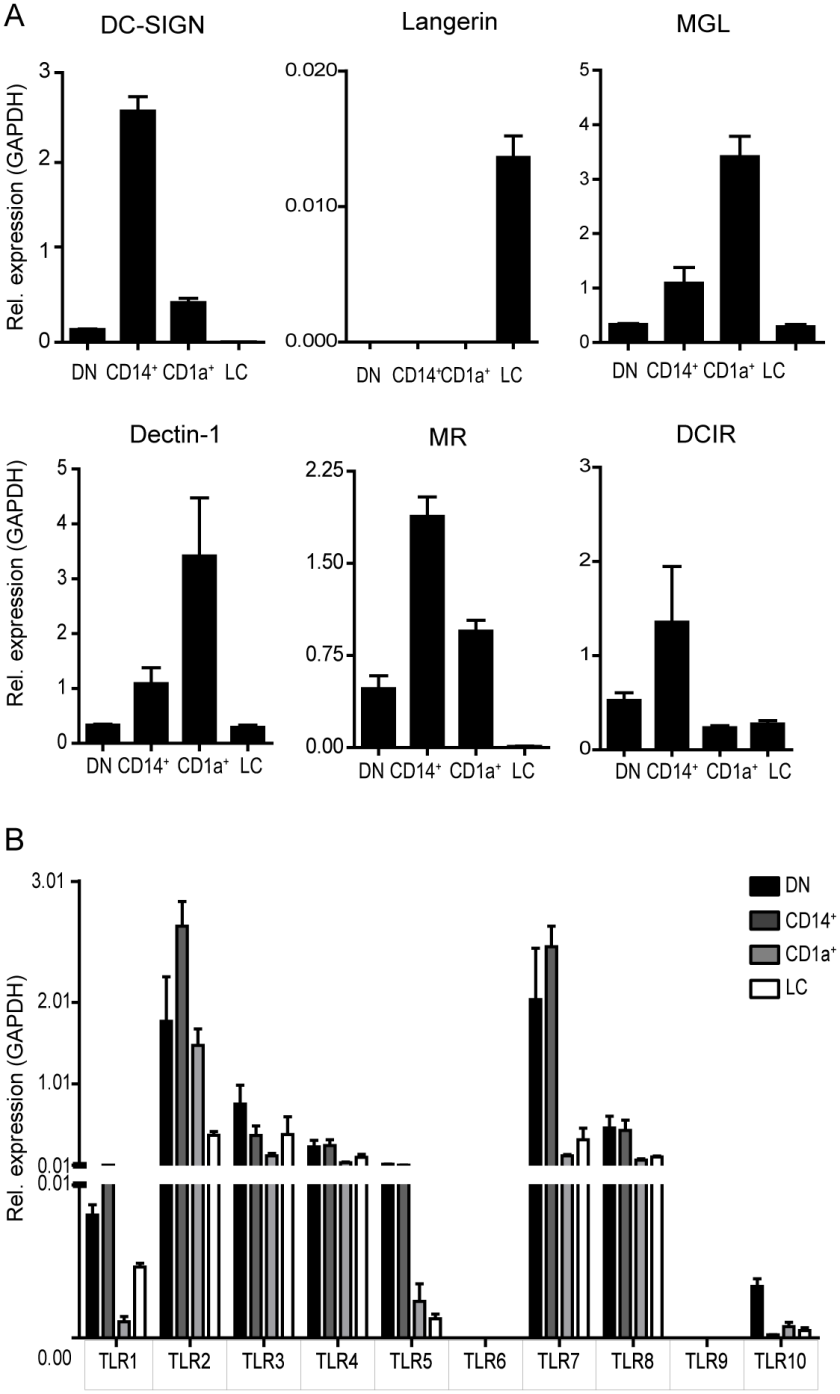
Expression of CLRs and TLRs by highly purified, FACS-sorted human skin APC subsets. mRNA expression of various CLRs (A) or TLRs (B) was examined on FACS-sorted human skin APC subsets using RT-PCR analyses. Due to low cell numbers of each subset after sorting, the distinct subsets contain combined cells of at least 4 skin donors. Mean values ± SEM; n = 4. Highly significant differences between the skin APC subsets were observed for all CLRs (p<0.001, as measured by the one-way ANOVA followed by the Bonferroni multiple comparison test).

In order to confirm the data obtained by the microarray analysis and to explore the expression pattern of CLRs and TLRs on the four separated skin APC subsets, we used real time PCR to analyze the expression of all human TLRs and a selected set of CLRs, which were all highly expressed by the dDCs, LCs or both in the microarray analysis. We performed the analysis on highly purified, FACS-sorted APC subsets, which enabled us to separate the dermal DCs in the three main subsets found under steady state conditions. CD14^+^ dermal cells expressed high levels of transcripts for DC-SIGN, mannose receptor (MR) and DCIR, whereas CD1a^+^ dDCs predominantly expressed MGL (Fig 3A). As expected, langerin transcripts were found only in human LCs, as shown by the microarray data (Fig 3A and Table 1). Dectin-1 transcripts were found in all four human DC subsets.

Transcripts for all TLRs, except TLR6 and TLR9, were expressed in all steady state skin APC subsets (Fig 3B). Interestingly, we could only detect minor differences that were not significant in TLR expression levels throughout the cell types investigated. Altogether, based on the expression of CD14, CD1a, CLRs, TLRs and co-stimulatory molecules 4 main population of human skin APCs can be operationally described: the relatively mature CD1a^+^ dDCs expressing MGL, high co-stimulatory molecules, the CD14^+^ dermal cells expressing DC-SIGN, DCIR and MR and CD86, the epidermal LCs expressing langerin and low co-stimulatory molecules and the DN dDC subset. The latter does not show specific markers, probably because this subset is most heterogeneous.

### Isolated APC subsets do not upregulate maturation markers after stimulation with TLR ligands

**Fig 4:**
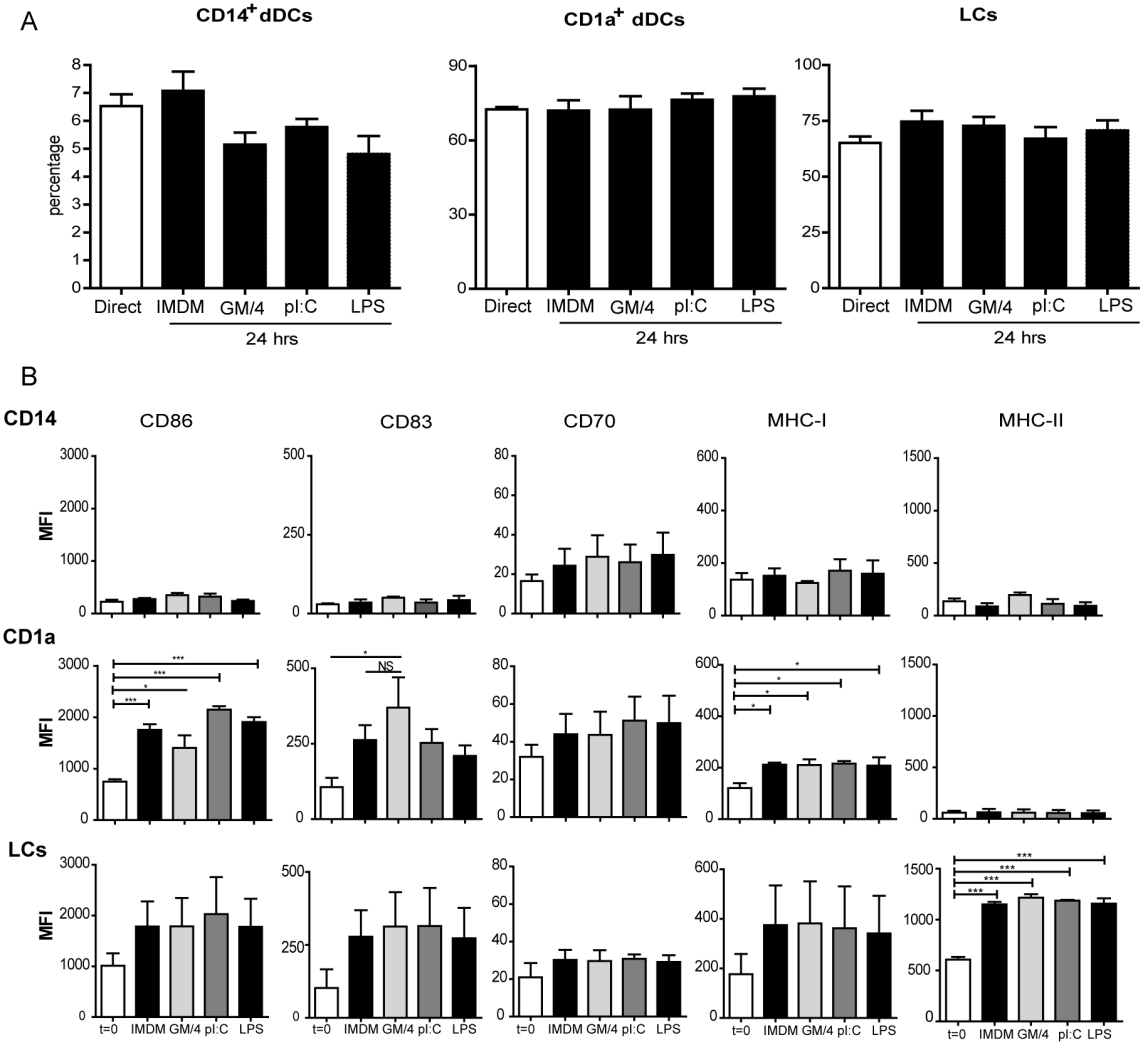
Human skin APC subsets do not phenotypically mature *in vitro* upon inflammatory conditions. A. Percentages of CD14^+^ dermal cells, CD1a^+^ dDCs and LCs (gated on HLR-DR^+^ cells) directly after enzymatic isolation or after 24 h of culture of epidermal and dermal suspensions in medium (IMDM), GM-CSF and IL-4 (GM/4), pI:C or LPS. Mean values ± SEM; n = 3. B. Surface expression of molecules associated with DC maturation, CD86, CD83 and CD70 and molecules association with T cell activation, MHC class I and II measured directly after enzymatic isolation of the skin APC subsets or after 24 h of culture of epidermal or dermal suspensions in the presence of indicated reagents. Mean values ± SEM; n = 3. *p<0.05 and ***p<0.001, as measured by the one-way ANOVA followed by the Bonferroni multiple comparison test.

In order to investigate whether TLR ligands activate freshly isolated APC subsets, we cultured the epidermal and dermal cell suspensions for 24 h *in vitro* in the presence of the TLR3 ligand pI:C, the TLR4 ligand LPS, or the cytokines GM-CSF and IL-4 (GM/4). Intradermal *ex vivo* injection of a combination of these two cytokines was previously shown to induce DC maturation [16]. LPS was used since it is a potent inducers of DC maturation in *in vitro* generated moDCs and intradermal application of pI:C had already been shown to enhance the maturation and T cell priming capacities of migratory skin DC[17]. No significant changes in the frequency of CD14^+^ dermal cells, CD1a^+^ dDCs or LCs were observed after 24 h of culture with GM/4, pI:C, LPS or medium as compared to untreated cells directly upon isolation (Fig 4A). These findings indicate that the skin APC subsets did not alter their subset characteristics or loss viability during the 24 h of *in vitro* culture despite of the different treatments. Next, we analyzed the expression of the maturation markers CD83, CD86 and CD70, as well as the expression of MHC-I and -II on the skin DC subsets. As shown in Fig 4B, the 24 h of culture in the presence of TLR ligands or GM/4 hardly affected the maturation status of any of the APC subsets. The CD1a^+^ dDCs showed the highest expression of co-stimulatory molecules, as previously observed under steady state conditions (Fig 2). LCs also displayed a certain level of spontaneous maturation after 24 h of culture in medium (Fig 4B), but as in the case of CD1a^+^ dDCs, the addition of GM/4, LPS or pI:C did not further enhance the maturation status nor were the CD14^+^ dermal cells affected in their maturation status after 24 h of culture. Together, these data provide evidence that isolated skin DC subsets do not respond to maturation signals by upregulating the surface expression of co-stimulatory and maturation molecules *ex vivo*.

### Isolated dermal APCs and LCs respond to TLR ligands by the production of pro-inflammatory cytokines and IL-10

**Fig 5:**
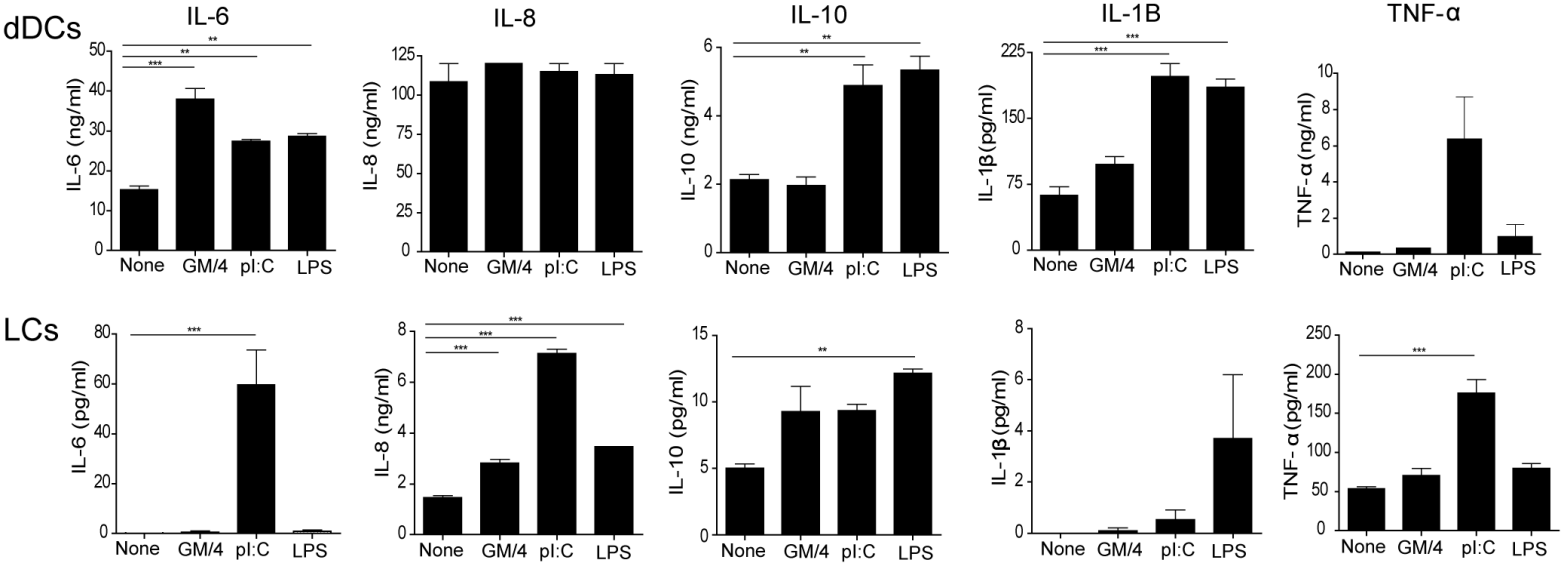
Dermal APCs and LCs secrete cytokines after culture in the presence of GM/4 or the TLR ligands pI:C and LPS. Enzymatically isolated LCs and dermal APCs were cultured for 24 h in medium or medium containing GM/4, LPS or pI:C. Secretion of cytokines was measured by ELISA. Mean values ± SEM; n = 3, each experiment measured in triplicate. *p<0.05, **p<0.01 and ***p<0.001 as measured by the one-way ANOVA followed by the Bonferroni multiple comparison test.

Although the dermal APC subsets and LCs did not mature phenotypically upon stimulation with the TLR ligands or GM/4, we also investigated the cytokine profile of these cells after 24 h of culture in the presence of IMDM, GM/4, pI:C or LPS. We compared the cytokine production of total dermal APCs and LCs and did not discriminate between the CD1a^+^ and CD14^+^ cells, since the separation of these subsets by MACS or sorting affected the dermal APCs substantially and hampered the cytokine production. In contrast to the lack of upregulation of co-stimulatory molecules observed in Fig 4, the dermal APCs responded to the TLR ligands with a significant increase in the secretion of IL-6, IL-1β, and IL-10 (Fig 5, upper panel). Additionally, secretion of TNF-α by the dermal APCs was enhanced after stimulation with pI:C (Fig 5). In contrast, LCs responded to pI:C by a significant increase in the secretion of IL-6, IL-8 and TNF-α as compared to medium alone, whereas LPS only induced significant changes in the secretion of IL-10 and IL-8 (Fig 5, lower panel). Overall, human LCs produced less cytokines compared to dermal APCs.

### CD14^+^ dermal cells have potent antigen internalization capacities, whereas LCs are most potent in antigen cross-presentation and CD8^+^ T cell activation

**Fig 6:**
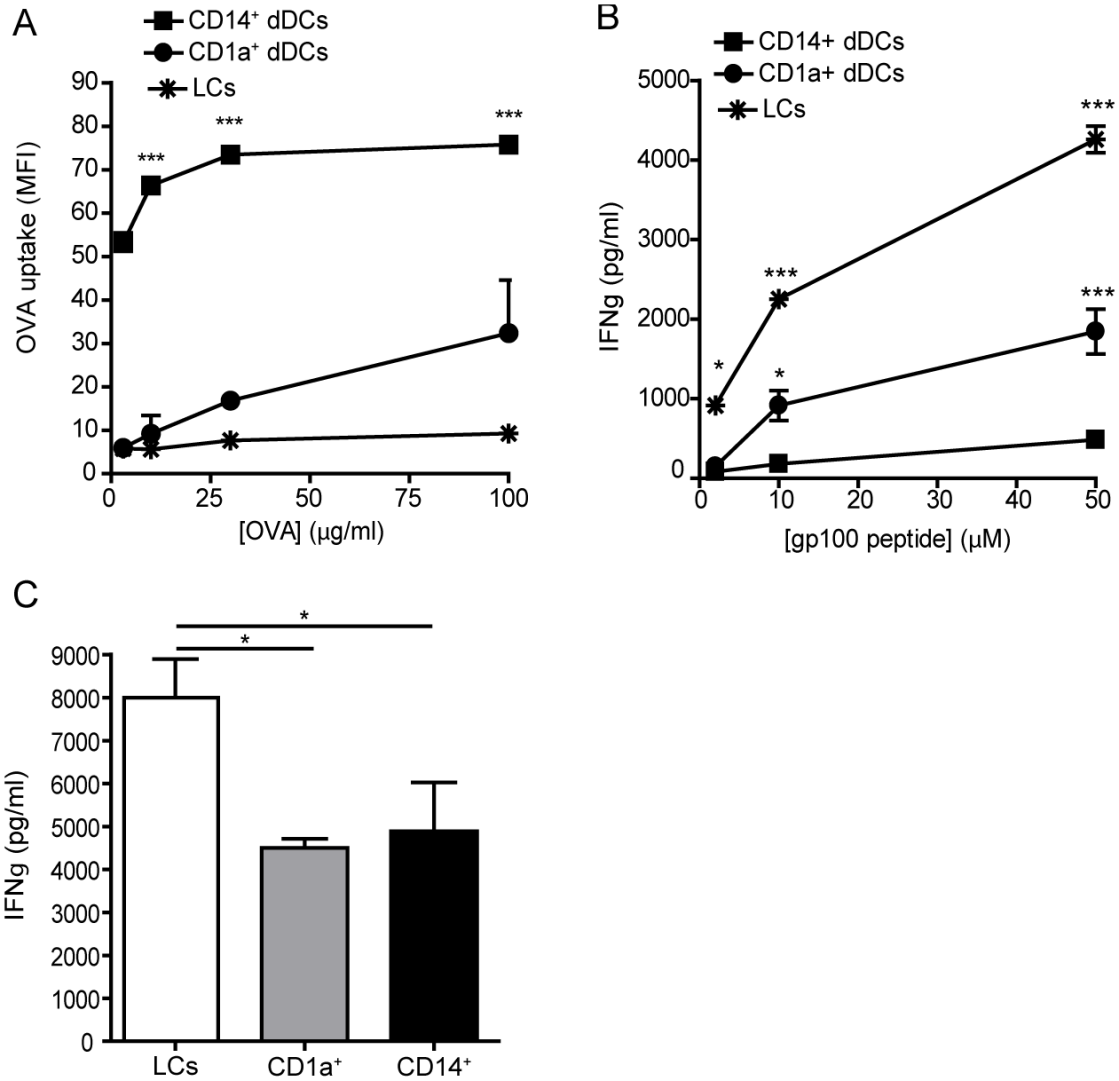
Antigen internalization and CD8^+^ T cell stimulatory capacities of steady state isolated human skin APC subsets. A. Internalization of fluorescently labeled OVA by the enzymatically isolated skin APC subsets after 2 h as measured by flow cytometry. A representative experiment is shown. Mean values ± SEM; n = 3. ***p<0.001 as measured by the two-way ANOVA followed by the Bonferroni multiple comparison test. B. Antigen-pulsed, MACS-isolated human skin APC subsets were co-cultured with HLA-A2^+^ GP100-specific CD8^+^ T cells. After 24 h, IFN-y levels were analyzed in the supernatants as measure for T cell activation using ELISA. A representative experiment is shown. Mean values ± SEM; n = 3, each experiment performed in triplicate. *p<0.05 as measured by the one-way ANOVA followed by the Bonferroni multiple comparison test. C. MACS-isolated human skin DC subsets were pulsed with the 9 aa long GP100 peptide for 2 h, washed, and co-cultured with the HLA-A2^+^ GP100-specific CD8^+^ T cell clone. IFN-y production by the T cells was measured using ELISA. Mean values ± SEM; n = 3, each measured in triplicate. *p<0.05 and ***p<0.001 as measured by the two-way ANOVA followed by the Bonferroni multiple comparison test.

To further characterize the functional properties of the isolated skin APCs, we analyzed the capacity of the cells to internalize and subsequently process antigens for presentation to CD8^+^ T cells. In particular DCs have shown to be superior in cross-presentation compared to the other APCs, an important mechanism to induce virus- or tumor-specific CD8^+^ T cells [2]. We exposed cells to fluorescently-labeled OVA (ovalbumin) and measured binding/uptake of OVA after 2 h using flow cytometry. The CD14^+^ dermal cells showed a significantly higher signal as compared to the CD1a^+^ dDCs and LCs (Fig 6A and S1 Fig). To determine whether the internalization of antigen also facilitated cross-presentation by the skin APC subsets, we allowed internalization of a 25 aa long GP100 peptide, which requires processing for loading on MHC-I molecules. The APC subsets were incubated with the 25 aa long GP100 peptide for 2 h and subsequently co-cultured with a CD8^+^ T cell clone specific for GP100_280–288_, an immunodominant epitope present in the 25 aa long GP100 peptide. As expected based on the maturation profile and antigen internalization data, CD1a^+^ dDCs showed a significantly higher dose-dependent activation of the CD8^+^ T cell clone compared to the CD14^+^ dermal cells, as measured by the production of IFN-γ (Fig 6B). Steady state human LCs were the most potent inducers of CD8^+^ T cell activation after cross-presentation (Fig 6B). Although steady state CD14^+^ dermal cells were very efficient at capturing antigen (Fig 6A), their cross-presentation capacity was rather poor (Fig 6B). To examine if LCs were truly superior in cross-presentation or whether the expression levels of MHC class I on these cells had any influence, we incubated the isolated skin APC subsets with the immunodominant peptide GP100_280–288_, which does not need processing and can be directly loaded on MHC class I molecules, and co-cultured the cells with the CD8^+^ T cell clone. This resulted in a significant higher activation of the T cell clone by the LCs compared to the CD1a^+^ and CD14^+^ dermal APCs, providing evidence that LCs have the highest ability to induce CD8^+^ T cell activation under steady state conditions (Fig 6C). In conclusion, although the CD14^+^ dermal subset internalized protein antigen most efficiently, the human LCs showed the highest capacity to cross-present peptide antigens and to activate CD8^+^ T cells.

## Discussion

Here we have shown that operationally distinct human APC subsets in the skin (based on CD14 and CD1a expression) in steady state (i.e. isolated *ex vivo*) display differential expression of CLRs and TLRs and cross-presenting ability. In addition, we have shown that the CD1a^+^ dDCs are the most mature human skin DC subset under steady state conditions based on the expression of co-stimulatory molecules and production of cytokines, in keeping with our earlier findings [24]. However, human LCs showed a superior ability to cross-present antigenic peptides compared to the CD1a^+^ dDCs and CD14^+^ dermal cells, based on the highest activation of antigen-specific CD8^+^ T cells. Gene expression profiling showed that human dermal APCs and LCs have different CLR signatures. We have found exclusive expression of DC-SIGN, DCIR, macrophage mannose receptor and CLEC1A on steady state dermal APCs. Most of these CLRs have a role in antigen internalization [25–27], illustrating that dermal APCs have a more specialized function in recognizing a broad repertoire of antigens for internalization than LCs. More specifically, the CD14^+^ APCs expressed higher levels of DC-SIGN, macrophage mannose receptor and DCIR compared to the CD1a^+^ dermal APCs (Fig 3A), which is line with our antigen internalization data presented in Fig 6A. Although the BDCA3^+^ dermal DCs are described as potent cross-presenting cells [13], we did not analyze them as a separate subset, because the BDCA3^+^ dermal DCs represent only a minority of the skin migratory cells. In addition, the double negative subset of dermal APCs, which contain the BDCA3^+^ dermal DCs, did not show a particular CLR expression pattern (Fig 3A).

For most experiments, we directly isolated human APC subsets using trypsin and collagenase instead of allowing the migration of the subsets. This method let us to analyze the phenotype and function of the subsets under steady state conditions. Spontaneous migration of skin APCs has been shown to already induce maturation and a reduction in antigen uptake capacities [28], thereby affecting both phenotype and function of the cells. Moreover, the migration protocol for skin APCs could potentially favor the migration of a particular subset, while non-migratory or slowly migrating cells could be excluded from analysis. Recently, it has been demonstrated that the pattern of gene expression by LCs and CD11c^+^ dDCs was retained when migratory skin subsets were compared to trypsinized subsets, despite the fact that phenotypic immunological maturation, i.e. increased expression of co-stimulatory molecules, was observed in the migratory skin subsets [29]. Understanding of the phenotype and function of both steady state and migratory skin APCs is necessary for the generation of targeted cutaneous vaccination strategies that induce immune stimulation, but need to prevent immune regulation. In addition, it has been shown that LCs induce activation and proliferation of skin-resident T regs under steady state conditions, whereas they activated and induced the proliferation of effector-memory T cells and limited T reg activation in the presence of foreign pathogens [30]. This indicate that LCs maintain tolerance in steady state healthy skin, but can activate protective skin-resident memory T cells upon infectious challenge. Therefore, studying skin APC subsets both in steady state and upon inflammatory stimulation is essential to design effective DC-targeting vaccination strategies.

In line with previously published work using migratory or *in vitro* generated LCs [8, 31–33], we here show that steady state LCs are superior in the cross-presentation of soluble peptides to effector-memory CD8^+^ T cells compared to dDCs. Although LCs were superior in the activation of CD8^+^ T cells, they produced significantly less pro-inflammatory cytokines (Fig 5) and were phenotypically less mature compared to steady state CD1a^+^ dDCs (Fig 2c). It has been suggested that LC maturation is tightly regulated by interactions with the microenvironment to prevent overt and harmful inflammatory responses, which may lead to disruption of the skin barrier and entry of infectious and harmful pathogens [29]. Moreover, we used memory T cell clones in our studies which are less dependent on co-stimulatory molecules and cytokines as compared to naïve T cells [27], which might also explain why steady state human LCs were superior in activating the Gp100-specific CD8^+^ T cell clone despite their low expression of co-stimulatory molecules and cytokines. Indeed in previous experiments, using separately migrated and cytokine-activated LCs and dDCs, we showed superior antigen-specific priming of naïve CD8^+^ T cells predominantly by CD1a^+^ dDCs in keeping with their phenotypically more mature phenotype [24]. The dissimilar methods used for the enzymatic isolation of human APC subsets, i.e. trypsin to isolate LCs and collagenase to isolate dermal APCs, might also differently affect the *in vitro* phenotype of LCs and CD1a^+^ dDCs. We cannot exclude that the differences in isolation methods might account for the finding that enzymatically isolated CD1a^+^ dDCs showed a more mature phenotype compared to the LCs. Here, we showed that enzymatically isolated LCs expressed low levels of maturation markers and hardly secreted pro-inflammatory cytokines upon TLR stimulation. However, migratory LCs responded to TLR3 stimulation by the production of TNF-α, IL-6 and the upregulation of CD86 and CD70 (data not shown).

Based on the high expression of multiple CLRs by the CD14^+^ dermal cells (Fig 3A) and their homology to murine CD11b^+^ macrophages [12], we assumed that these cells would internalize antigens efficiently. Indeed, the data presented in Fig 6A demonstrated that CD14^+^ dermal cells internalized significantly more OVA compared to CD1a^+^ dDCs. A possible explanation could be that CD14^+^ dermal cells are more efficient in receptor-mediated antigen internalization, facilitated through the highly expressed CLRs DC-SIGN and MR, which is in line with previous findings were we have shown that the migrated CD14^+^ cells internalized more Dextran-FITC compared to the CD1a^+^ dDCs [11]. In addition, DC-SIGN-mediated internalization of glycan-modified liposomes by CD14^+^ dermal DCs resulted in enhanced cross-presentation compared to the CD1a^+^ dDCs, providing evidence that the antigen formulation and mode of internalization have an influence on antigen cross-presentation [33, 34]. The formulation of the antigen has also been shown to be important for LC-mediated antigen cross-presentation. In the work presented here we describe increased antigen cross-presentation by LCs compared to the two dermal APC subsets after internalization of a soluble long peptide *in vitro*. Others reported a lack of antigen cross-presentation by human LC when the cells were targeted with glycan-modified liposomes[34] or inactivated measles virus *in vitro* [35]. In mice, the relevance of LCs to CD8^+^ T cell-mediated immunity is still under debate. In particular, murine LCs were shown to be dispensable over the langerin^+^CD103^+^ dermal DCs for the induction of CD8^+^ T cell responses in several models of viral infections, tumor or self-antigens [36–38], while some studies described LCs as essential for protective CD8^+^ T cell-mediated immunity [39–41]. Recently, is has been described using a transcriptional profiling approach combined with computational analyses and functional assays that the function of LCs may not be conserved between mouse and human [42]. Interestingly, human LCs and mouse XCR1^+^CD8α^+^CD103^+^ DCs, known for its superior cross-presentation, shared transcriptional modules containing genes related to MHC-I-mediated antigen processing and cross-presentation, which were not seen in mouse LCs [42]. Further studies are required to investigate the potential of human LCs to cross-present antigen *in vivo* and to define the conditions which favor LC-mediated cross-presentation *in vitro* and *in vivo*.

A lot of research has focused on the comparison between LCs and total dermal APCs, without making a distinction between the CD14^+^ and CD1a^+^ dermal cells, showing that LCs are phenotypically and functionally more mature than dDCs [24, 43, 44]. We previously performed a genome-wide analysis between isolated steady state LCs and CD1a^+^ dDCs and reported superior activation of CD1a^+^ dDC in keeping with our findings in the present study, similarly showing CD1a^+^ dDCs to be more mature compared to LCs under steady state conditions, as measured by maturation markers and pro-inflammatory cytokine production. Since CD1a^+^ dDCs represent a large proportion of relatively mature dDCs with high expression levels of CCR7 suggesting a high migratory potential to draining lymph nodes [12], more research should be aimed at further investigating these cells in DC-targeted vaccination protocols to induce (anti-tumor) immune responses. Importantly, in this study we additionally analyzed the distinct CD14^+^ and CD1a^+^ subsets of dermal APCs with emphasis on CLR and TLR expression patterns. We recently demonstrated that the unique expression of langerin on LC and DC-SIGN on CD14^+^ dermal cells, can be used to enhance receptor mediated antigen uptake and cross-presentation in these cells [33]. Importantly, we have demonstrated that the size and or multivalency of the targeting structure is of crucial importance to facilitate targeting through DC-SIGN or langerin and cross-presentation by the CD14^+^ dermal cells and LCs, respectively [33].

We have found that the isolated LC and dermal APC subsets were not sensitive to *in vitro* TLR stimulation at concentrations where moDCs responded vigorously showing an upregulation of co-stimulatory molecules (Fig 4B). Rather, we found that culturing both freshly isolated LCs and dermal APCs overnight in medium without activating cytokines or TLR ligands was already enough the induce an upregulation in the expression of co-stimulatory molecules similar to the upregulation seen when TLR ligands were added to the cultures. No changes were observed in the percentages of LCs, CD14^+^ cells or CD1a^+^ dDCs after overnight culture with cytokines or TLR ligands, suggesting that the subset distribution under these *in vitro* culture conditions was stable. This relative unresponsiveness of dDCs and LCs to TLR ligands might be induced by factors still present after isolation from the skin and is in line with our previous findings in the migrated cutaneous DC subsets after intradermal administration of TLR ligands [16]. In both studies we observed modest activating effects of TLR3 engagement by pI:C. In previous studies, LCs were described to express TLR6 and a very low or undetectable expression of TLR4 [45, 46]. However, we could detect mRNA of TLR4, but not of TLR6 expressed by isolated human LCs (Fig 3B). This discrepancy could possibly be explained by the use of different cell subsets to which the expression levels of the LCs are compared. Van der Aar and colleagues compared total dermal DCs with LCs, whereas we compared the three main subsets of human skin APCs [45]. Flacher *et al* compared expression levels of TLRs between LCs, keratinocytes and two subsets of blood DCs, but did not made any comparison between LCs and dermal DC subsets [46].

Altogether, our data clearly showed that the 3 main populations of APCs present in the steady state human skin each express a different set of TLRs, CLRs, co-stimulatory molecules and display a distinct CD8^+^ T cell cross-presenting capacity. Steady state CD14^+^ dermal cells are less mature cells with a high capacity to internalize antigens, whereas the LCs were superior in the activation of CD8^+^ T cell responses. Based on these results, LCs and CD1a^+^ dDCs seems suitable targets for targeted anti-tumor immunotherapy using soluble peptides in the presence of TLR3 ligands.

## Supporting Information

S1 Fig Antigen internalization by steady state human skin APC subsets. Internalization of fluorescently labeled OVA by the isolated skin APC subsets after 2 h as measured by flow cytometry. Data of one representative experiment are shown (n = 3). Filled histograms: unstained APCs, line histograms: OVA-AF549.
(PDF)

## Abbreviations

APC: Antigen presenting cells
CLR: C-type lectin
DC: Dendritic cell
DCIR: Dendritic cell immunoreceptor
DC-SIGN: Dendritic Cell-Specific Intercellular adhesion molecule-3-Grabbing Non-integrin
Tfh: Follicular helper T cell
HLA: Human leukocyte antigen
IL: Interleukin
LC: Langerhans cell
LPS: Lipopolysaccharide
MMR: Macrophage mannose receptor
MHC: Major histocompatibility complex
moDC: Monocyte-derived dendritic cell
pI:C: Polyribosinic-polyribocytidylic acid
Tregs: Regulatory T cells
TLR: Toll-like receptor
