# Supplementary material for: Phenotypic and Functional Properties of Human Steady State CD14+ and CD1a+ Antigen Presenting Cells and Epidermal Langerhans Cells
Source: PLoS One. 2015 Nov 25;10(11):e0143519. doi: 10.1371/journal.pone.0143519 (PMC4659545; doi:10.1371/journal.pone.0143519)
Supplement: S1 Fig — Internalization of fluorescently labeled OVA by the isolated skin APC subsets after 2 h as measured by flow cytometry. Data of one representative experiment are shown (n = 3). Filled histograms: unstained APCs, line histograms: OVA-AF549. (PDF) [file pone.0143519.s001.pdf]

S1 Supporting information.

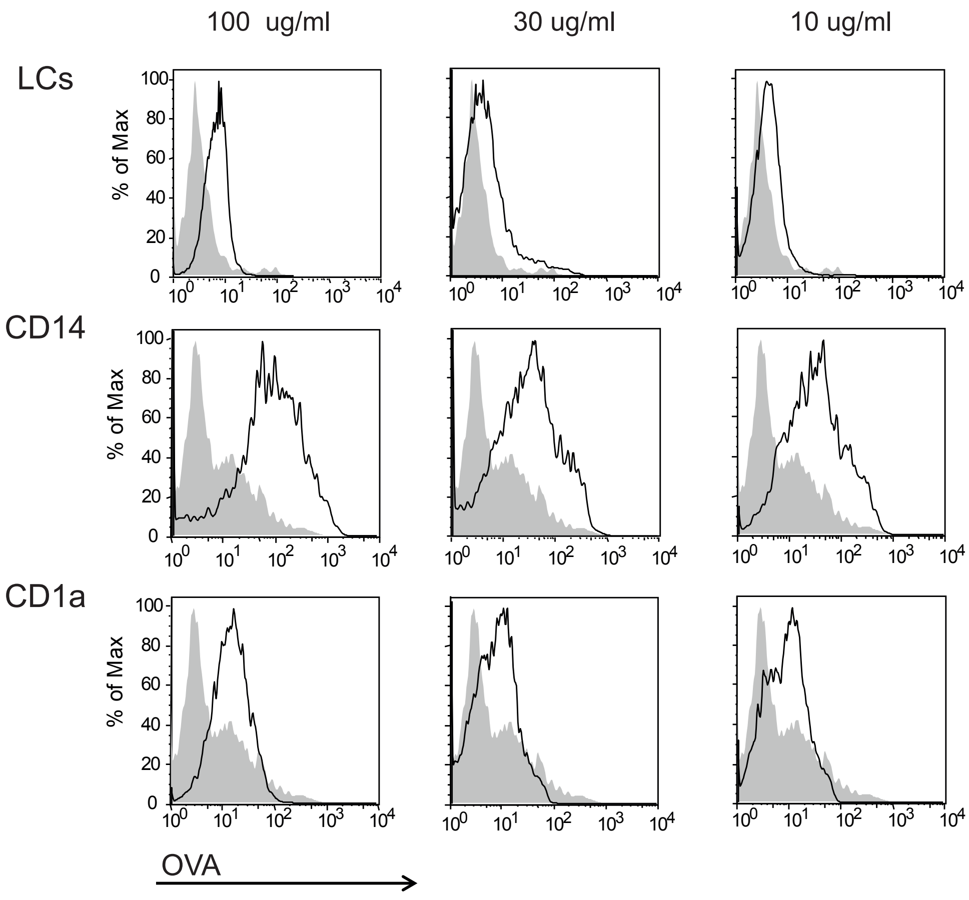

S1 Fig 1. Antigen internalization by steady state human skin APC subsets.
Internalization of fluorescently labeled OVA by the isolated skin APC subsets after 2 h as measured by flow cytometry. Data of one representative experiment are shown (n=3). Filled histograms: unstained APCs, line histograms: OVA-AF549.
